# Annotations

**Published:** 1901-05-04

**Authors:** 


### ANNOTATIONS.

### The Components of Fruit Wines.

An interesting prosecution which has recently taken place in Liverpool may make people think twice before they purchase what are called fruit wines. These wines are non-alcoholic, and failing spirit something must be put into the fruit juice to preserve it. The something is salicylic acid. The Health Committee of Liverpool instituted a prosecution against Messrs. Paterson, manufacturers of fruit wines, for having introduced into their wines an amount of salicylic acid which was dangerous to health. One of the committee's inspectors purchased a bottle of ginger wine made by this firm, which contained the acid in the proportion of 13 grains to the pint. Mr. Collingwood Williams, the public analyist of Liverpool, gave it as his opinion that one grain of salicylic acid per pint was enough for a preservative, and Professor Boyce, of University College; Professor Paul, surgeon at the Royal Infirmary; and Dr. Guinbaum, of University College, united in declaring that the amount of the acid in the defendants' wines was injurious to the human system. On the other hand, Dr. M'Allister, hon. physician to the Royal Southern Hospital; Dr. Bradshaw, and Dr. Bickerton declared that salicylic acid in the proportion found in Messrs. Paterson's wines was absolutely harmless, and the last-named gentleman added the testimony that he and his family had used these wines regularly for months and were none the worse. Mr. Tobin, the defendants' counsel, explained that they had made experiments to find out how little salicylic acid was necessary to preserve the fruit juice, but the result had been to prove to them that to reduce the quantity by even 25 per cent. would make it impossible for the wines to keep. Twice before have they been prosecuted on account of the amount of acid in their wines, but have been acquitted on both occasions, and this time also the stipendiary magistrate, Mr. Stewart, came to the conclusion that "drunk to the extent which it was reasonable to suppose such a beverage would be taken" the amount of salicylic acid would not be harmful to the consumers. He therefore held that the Committee had not proved their case, and gave judgment for the defendants. He refused, however, to give costs, as the Health Committee had done only their duty in seeking to have the matter investigated. "He was afraid that Messrs. Paterson must be content with the excellent advertisement." But is it such an excellent advertisement after all?

### Rats and Plague.

A timely memorandum on the subject of the diffusion of plague by rats has just been issued by the Medical Department of the Local Government Board, for the guidance of the captains of ships and of the sanitary authorities of our seaports. The well-known liability of the animals to contract the disease, and the share which they have undoubtedly had in its diffusion, are succinctly set forth; and it is recommended that means should be taken to render difficult the access to shipping of shore rats in infected places, and also to destroy rats, either on board ship or on shore, by whatever means may be available for the purpose. These means are not specified in the memorandum, and are, presumably, to be left to the members of the rat-destroying profession; but there is specific advice to protect ships by placing guards on their cables and hawsers, so that these may not be converted into lines of route by invaders. It is also directed that the carcases of dead rats should be consumed by fire with as little handling as possible. The memorandum is one the importance of which should not be underestimated. We do not very often see rats, and have probably no adequate idea of their numbers. Among the many charming papers written by the late Richard Jefferies, there was one on "shy animals," in which he described woodland creatures which, because they are seldom seen, are supposed by most people to be scarce, but which, he said, are really more numerous in England than men, women, and children. There is every reason to believe that the results of an accurate census of rats would require a very formidable array of figures for their correct expression; and we are not aware of any useful work which rats accomplish, or of any evils to be apprehended from their extinction. In some countries, no doubt, they may do good as scavengers; but their services in this direction can hardly be required in the days of universal sewerage, and of the systematic utilisation or disposal of refuse.

